# Research on the frictional contact behaviors of high-speed motorized spindle bearing with oil-air lubrication

**DOI:** 10.1038/s41598-026-39860-2

**Published:** 2026-03-21

**Authors:** Weitao Jia, Junhao Guan, Feng Gao, Xian Wei

**Affiliations:** 1 School of Mechanical and Electrical Engineering, Zhengzhou University of Technology, Zhengzhou, China; 2 Henan Shijia Photons Technology Co. LTD, Hebi, China; 3 Sanmenxia Huayi Machinery Co. LTD, Sanmenxia, China; 4 School of Mechanical and Precision Instrument Engineering, Xi’an University of Technology, Xi’an, China; 5 College of Intelligent Manufacturing, Panzhihua University, Panzhihua, China; 6 Sichuan Engineering Research Center for Titanium Alloy Advanced Manufacturing Technology, Panzhihua, China

**Keywords:** Oil-air lubrication, Point contact pair, Friction coefficient, Temperature rise, Wear rates, Mechanical engineering, Structural materials

## Abstract

As an important supporting element of rotating mechanisms, the frictional wear of rolling bearing causes low motion accuracy. To simulate the service statuses of rolling bearing under different lubrication conditions, the wear and frictional behavior between the GCr15 ball-on-disc contact pairs was experimentally analyzed to investigate the effects of rotating speed, loading, supplied oil quantity and air pressure on the friction and wear properties of the point contact pairs. As the experimental results show, the use of oil-air lubrication yields the smallest friction coefficient as compared to those of the grease and the oil lubrication modes, which can suppress the temperature rise effectively and attain the lowest wear rates. The increase of oil volume fraction for the oil-air lubrication led to diminished wear scar depth and low wear rates, while the supplied oil quantity and air pressure were constant, the increase of rotating speed resulted in slight decline of friction coefficient, as well as gradual elevation of temperature and wear rates. Regarding loading, its enhancement made the friction coefficient and wear scar depth went up initially and then reduced, which also caused the wear rates decreased. The findings of this research provide a useful basis for selecting the optimal high-speed bearing lubrication method and their operating parameters.

## Introduction

In mechanical transmission, rolling bearing is often used as the supporting element for rotating shafts owing to their low friction coefficient and high transmission efficiency. Given the larger loading capacity per unit area of point contact between the ball and the raceway, wear is easily produced during relative sliding^1–3^, which is a major cause of lowered rotation accuracy and compromised system reliability. To overcome the negative effects caused by frictional wear, reasonable lubrication of bearing is a highly feasible means. Grease lubrication, oil lubrication or oil-air lubrication are generally applied to lubricate bearing. The frictional wear performance of contact pairs is dependent on the distribution of lubricating medium^4^. In the research of bearing lubrication performance, single-phase method is adopted in general. Grease is mostly used in the condition of being difficult to continuously supply lubricating medium. The steady-state temperature distribution of bearing systems and the microstructure of fretting wear scars on bearing surface can characterized the performance of grease lubrication^5–8^. Oil lubrication is another traditional single-phase lubrication. Various operating conditions had a significant influence on the bearing lubrication characteristics during using oil lubrication^9–12^. Compared to the grease and oil lubrication modes, with oil-air lubrication, the compressed air can carry lubricating oil to the lubrication region to form a continuous oil film. In the meanwhile, it takes away substantial frictional heat, thereby achieving good cooling and lubricating effects^13–16^.

In order to observe the moving status of the ball in the raceway in detail, the rolling-sliding friction testing methods are mostly employed. At present, there are two mainstream modes used for investigation of rolling contact fatigue status for ball bearing, namely the “rolling-sliding disc” test (deals with linear contact problems), and the “ball-on-disc” test (deals with point contact problems)^17^. Wang et al. studied the tribological property of ATG composite ceramic material during sliding on a GCr15 disc, and discussed the effects of sliding speed and normal load on the friction coefficient and wear rates. They found that the friction coefficient increased with the enhancing normal load, while decreased with the heightening sliding speed^18^. According to ball-on-disc experimental studies concerning the friction properties of point contact pairs under oil lubrication, the increase of rotating speed led to a gradual decrease of friction coefficient, which tended to keep at a stable state^19–22^. Based on a numerical approach^23^, Pei et al. explored the variation exhibition of friction coefficient during rolling contact wear under oil lubrication, which revealed that the friction coefficient dropped rapidly initially and then remained almost unchanged during the wear process^24^. Through frictional wear experiments, Zeng et al. obtained the average friction coefficient of friction pairs under oil lubrication, where NiTiNOL60 alloy pin slid back and forth on GCr15 steel disc. Their results demonstrated that under oil lubrication, oil film was easily formed on the frictional contact surface, so that the friction coefficient was reduced and remained stable for a relative long period^25^. Liu et al. performed a sliding friction experiment on the oil-air lubrication point contact pairs in view of the dual advantages (cooling and lubrication) of the oil-air lubrication, and compared the results with the dry friction and drip lubrication experiments in the same operating conditions. Their study revealed that under oil-air lubrication, the increase in supply oil quantity led to downward trends in the friction coefficients and wear scar parameters. After the supply air velocity reached a certain value, the oil-air lubrication attained lower friction coefficient and milder morphological variation of wear scar than the drip lubrication^26^. Through an experiment for point contact sliding frictional wear under oil-air lubrication, Liu et al. probed into the impact of air velocity on the point contact sliding wear characteristics. They found that the air velocity was significantly influential to the point contact sliding wear. At a proper air velocity, the volume fraction of oil increased with the growing supply oil quantity, and the friction pair presented the best lubrication performance. As a result, the appropriate values of oil-air pressure and oil volume fraction were attainable in the vicinity of the point contact zone, which was conducive to diffusing the fine oil droplets, and to reducing the sliding friction coefficient and wear rates^27^. According to an oil-air lubrication experiment performed by Wu et al. on the ball-on-disc point contact pair, the friction coefficient tended to drop initially and then rise with the increasing supply of air velocity^28^. To attain the best lubrication effect, the oil-air lubrication systems need to adopt different supply oil quantity designs depending on the operating conditions. The effects of supply oil pressure on the force and friction coefficient for sliding bearing were explored by Ali Ahmad^29^, through which it was found that the friction force and coefficient of fluid are affected to some extents by the change of supply oil pressure. Liu studied the lubrication behavior of micro-oil droplets passing through the elastohydrodynamic lubrication contact region, and the influence of load on the lubrication behavior was also investigated. This article proposes a fluid dynamic pressure friction model to predict the friction coefficient of bearings. Experiments show that the predicted friction coefficient of bearings by the fluid dynamic pressure friction model is closer to the actual value than the dry friction model^30^. This study aims to investigate the influence of rare earth addition on the friction and wear behavior of M50 bearing steel. The results show that rare earth addition has little effect on friction coefficient, but can reduce wear volume especially under harsh wear conditions^31^. Effect of choline chloride-multiple diols deep eutectic solvents on the friction and vibration performance of Si_3_N_4_-GCr15 hybrid ceramic ball bearings is studied, the results indicate that friction coefficient, wear scar diameter, bearing vibration, and friction torque decreased, respectively^32^. The prediction of the sliding wear on the surface micro-topography coupled with the material hardness and wear time bearings is proposed, this method provides a valuable tool for the evaluation of lubrication performance^33^. This paper carries out experimental research on the bearing cage mass imbalance working condition, and elucidates the wear characteristics and mechanisms of high-speed angular contact ball bearings under the condition of cage mass imbalance, so as to provide a theoretical basis for the reliability design of the bearing of high-speed machine tools^34^. As their results demonstrated, the spreading process of oil droplets was impacted by load, with greater load indicating less spreading of oil droplets and the initial spread position closer to the contact zone.

Most of the foregoing studies concerning frictional wear of oil-air lubricated point contact pairs focused on the conditions of sliding and low-speed rolling friction. Given the generally high-speed of ball bearing, however, it is of profound practical significance to research the effects of frictional wear behaviors of high-speed point contact pairs under oil-air lubrication. In this paper, through comparisons with friction experiments under the grease and oil lubrication, the influences of operating condition parameters on the friction coefficient, temperature rise and wear rates of point contact pairs are explored, and the optimal lubrication method and corresponding parameters are identified, thereby achieving improvement of motion accuracy and extension of service life.

## Experimentation

### Methodology

**Fig. 1:**
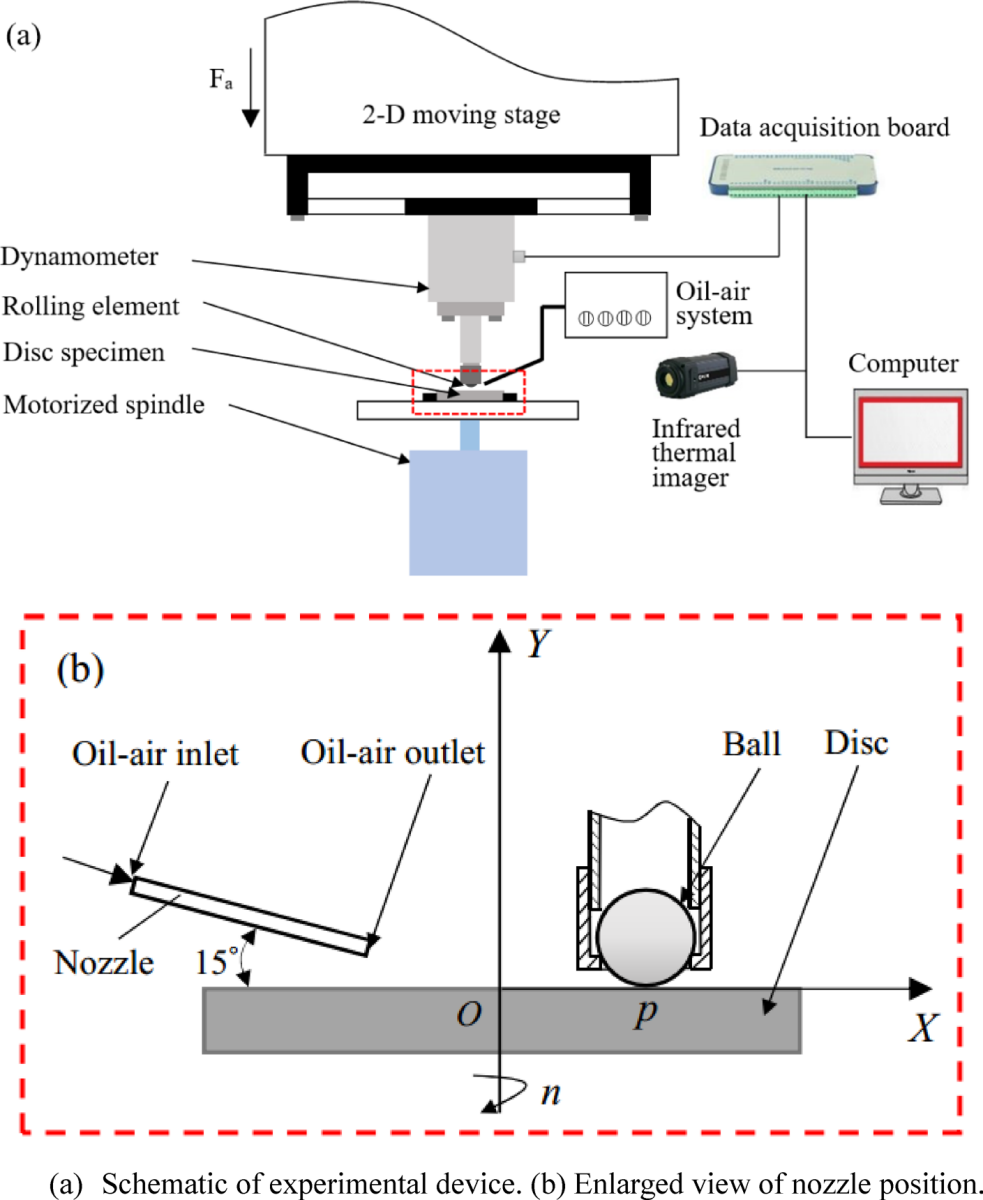
Experimental device.

In Fig. 1(a), the working principle of the ball-on-disc friction test device is illustrated under the condition of oil-air lubrication. The steel ball was restricted with a nut, the holder was bolted to the bottom of dynamometer, and the disc sample was fixed on a friction disk that rotated at a high-speed. During the experiment, the steel ball contacted the disc sample and was pushed by the preset load. The coordinate origin *O* was located at the upper surface center of the disc sample, *X* and *Y* were the horizontal and vertical coordinate, and the *Z* axis was perpendicular to the *XOY* plane pointing outwards. The through hole of the nozzle had an inner diameter of 2.5 mm, whose axis was within the *XOY* plane, and intersected with the upper surface of disc sample at point *p*, Fig. 1(b) displays the installation layout of the nozzle.

### Materials and operating condition parameters

In Table 1, the mechanical property parameters of the steel ball and disc sample used in the present experiments are detailed. Prior to the experiments, the disc sample were sanded sequentially with 400^#^, 600^#^ and 800^#^ water-resistant alumina sandpaper to remove surface burrs, which were then polished on the MP-2 polishing machine with metallographic sandpaper and polishing cloth to attain the surface roughness of 0.06 μm and the surface hardness of 60HRC. Afterwards, the sample were ultrasonically cleaned in acetone, ethanol solution and distilled water sequentially, followed by blow drying with a dryer.

**Table 1:** Mechanical property parameters of frictional pair materials.

Sample	Material	Elastic modulus(GPa)	Surface hardness(HRC)	Roughness	Possion’s ratio	Sizes(mm)
Ball	GCr15	208	62	Ra0.06	0.3	Φ7.94
Disc	GCr15	210	60	Ra0.06	0.3	Φ29 × 4

In Table 2, the experimental condition parameters are described. (1) During the grease lubrication experiment, the grease was spread evenly onto the sample surface. Table 3 lists the physical property parameters of lubricating grease. (2) In the oil lubrication experiment, the oil was dropped onto the disc sample surface with a rubber dropper. Table 4 lists the physical property parameters of lubricating oil. (3) During the oil-air lubrication experiment, the mixture of oil-air produced by the minimal oil-air lubrication system was sprayed onto the disc sample surface by high pressure.

**Table 2:** Test operating condition parameters.

Experimental parameters	Parameter value
Radius of ball rotation (mm)	6
Rotating speed(r/min)	2000/3000/4000/5000
Supply oil quantity(mm^3^/h)	30/37/51/65
Supply air pressure(MPa)	0.15 ~ 0.3
Load(N)	10/15/20/25
Lubrication modes	grease lubrication/oil lubrication/oil-air lubrication

**Table 3:** Physical properties parameters of fag Arcanol-L75 SPEED2.6 grease.

Viscosity(40℃) (mm^2^/s)	Density(20℃) (kg/m^3^)	Working temperature (℃)	Continuous work limit temperature (℃)	Speed limit (mm/min)	Worked penetration (mm)	Drop point (℃)	Additive
22	9.4 × 10^2^	- 40 ~ 120	80	2 × 10^6^	250 ~ 280	> 250	T351

**Table 4:** Physical property parameters of 46^#^ mechanical oil.

Viscosity(40℃) (mm^2^/s)	Flash point (℃)	Pour point (℃)	Density(20℃) (kg/m^3^)	Surface tension(25℃) (*N*/cm)	Specific heat(100℃) (J/(kg·K))	Mechanical impurities (%)	Additive
43.2	2.18 × 10^2^	- 27	8.61 × 10^2^	27.5	0.56	0.007	ZDBDP

### Experimental procedure

**Fig. 2:**
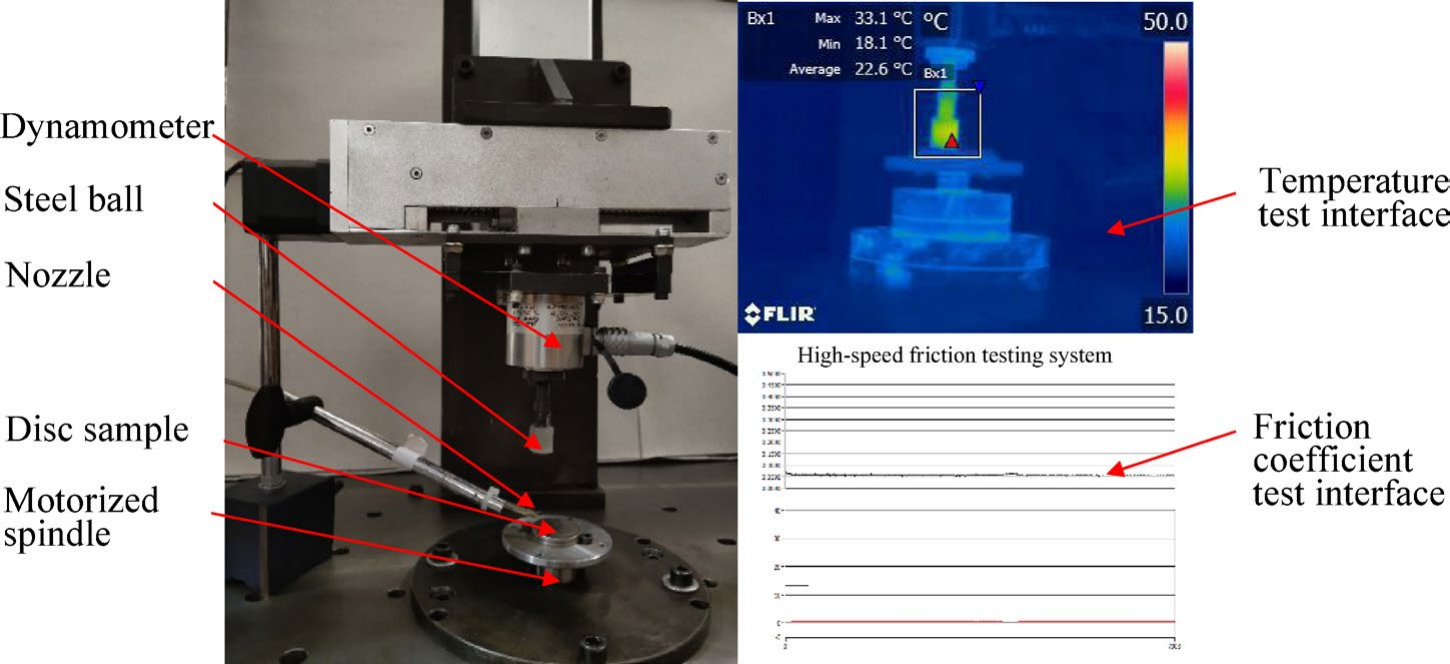
High-speed friction test device.

As shown in Fig. 2, the rotating action and the loading applied by the ball of the friction disc were implemented by a motorized spindle and a servo motor loading device respectively. The ball was fixed to the end of loading rod via a nut, whereas the disc sample was fastened by a three-jaw chuck on the rotary table. By adjusting the loading stroke, the pressure of ball-on-disc contact pair was set the predefined normal load, and relative friction was generated along with the rotation of disc sample. Place the samples in acetone, ethanol solution, and distilled water in sequence, clean them using an ultrasonic cleaner, and dry them with a fan. For different lubrication methods, when using grease lubrication, first apply a layer of lubricating grease with a thickness of 0.5 mm on the surface of the disc sample before testing; when lubricating with oil, apply 0.05 ml of lubricating oil on the surface of the disc sample before testing; during oil-air lubrication, oil is continuously provided by the oil-air lubrication equipment. Through this device, the friction coefficient and temperature between friction pairs under the action of different lubricants can be quantitatively measured. The friction forces in the *X*, *Y* and *Z* directions were obtained using the USB2884 PC-DAQ made by ART Technology Co. Ltd., by which the friction coefficients were computed. The FLIR-A315 infrared thermal imager was utilized to collect the temperature of the friction pairs, which was used as the main basis for evaluating the lubrication effect. Before the experiment, in order to reduce accidental errors, verify equipment performance, and optimize the operation process, multiple repeated experiments were conducted, and the experimental platform was adjusted to ensure data consistency. According to the pre-test results, the test duration is one hour, and the fluctuation range of friction coefficient, temperature rise is less than 1%, therefore, one hour is taken as the test duration. During the experiment, the room temperature was maintained around 20℃.

## Results and discussions

### Frictional wear properties and temperature rise

**Fig. 3:**
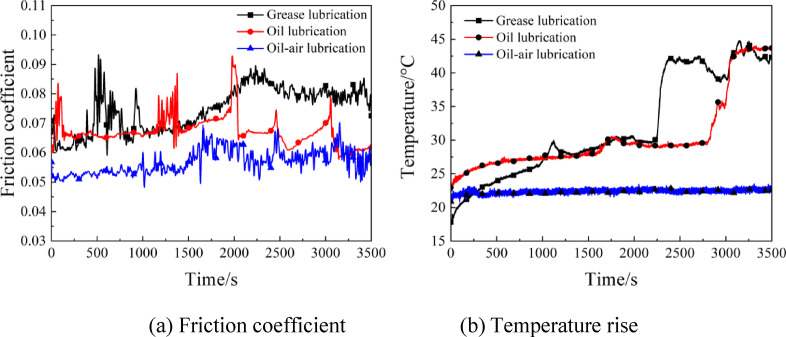
Effects of lubrication modes on the friction coefficient and temperature rise.

In Fig. 3, the experimentally measured variations of friction coefficient and temperature rise over time are depicted under grease, oil and oil-air lubrication at rotating speed of 4000r/min and load of 15 N. As is clear from Fig. 3(a), the friction coefficient fluctuated greatly during the running-in period(0 ~ 2000s), which then tended almost to be unchanged after entering the steady wear stage(2000 ~ 3500s). In the case of grease lubrication, the friction coefficient presented an upward then downward trend, which eventually stabilized at 0.078. Under oil lubrication, the friction coefficient oscillated little during the running-in period. With the oil-air lubrication, the friction coefficient presented an upward trend, and the steady-stage friction coefficient was the lowest as compared to the former two lubrication methods, with a value of 0.055. The explanation for this phenomenon is that during grease lubrication, the lubricating grease had a poor fluidity since it was in a semi-solid state, which was easily squeezed out of the contact zone by fretting action. In certain conditions, the lubricating grease neighboring around contact zone was taken into the zone by the relative motions between ball and disc, which resumed the lubricating oil film to relubricate the contacted pair, thereby leading to the small-range fluctuation of friction coefficient. During oil lubrication, the lubricating oil drips could be delivered accurately and adhered to the friction pairs due to the lubricant being not interfered by compressed air, exhibiting a relatively preferable lubrication effect. As for the case of oil-air lubrication, the two-phase flow of oil-air was ejected outwards via the nozzle end, and the lubricating oil reached the space between friction pairs continuously and evenly, thereby forming a satisfactory lubricating oil film. From Fig. 3(b), it is clear that: (1) With grease lubrication, the temperature of friction pairs rose ceaselessly with the prolonging operating time. After running for 2300s, the temperature was as high as 42℃, which then tended to stabilize. (2) Under oil lubrication, the temperature of frictional contact zone rose initially and then stabilized with a steady-stage temperature of 44℃. (3) The use of oil-air lubrication led to a marked drop in the temperature of frictional contact zone, and throughout the experiment, the contact zone temperature was lower than 23℃. Due to the continuous supply of compressed air and lubricant by the system, a good oil film is formed, effectively isolating the metal surface and reducing direct contact friction, the bearing temperature was rather low, which is thus suitable for the lubrication of high-speed friction pairs.

**Fig. 4:**
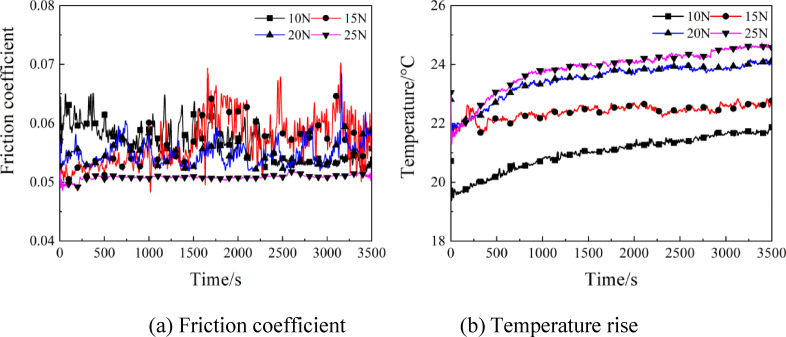
Effects of load on the friction coefficient and temperature rise.

**Fig. 5:**
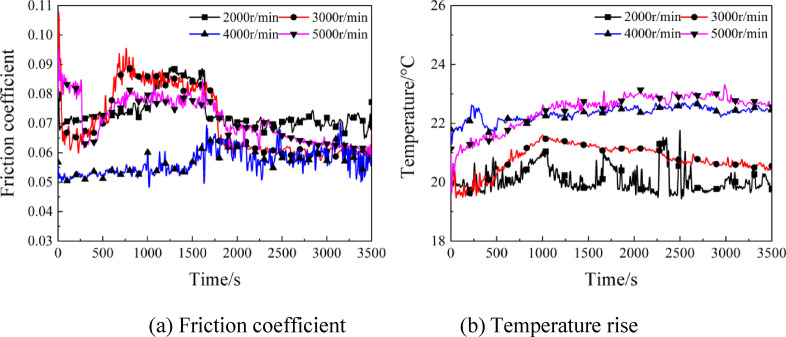
Effects of rotating speed on the friction coefficient and temperature rise.

**Fig. 6:**
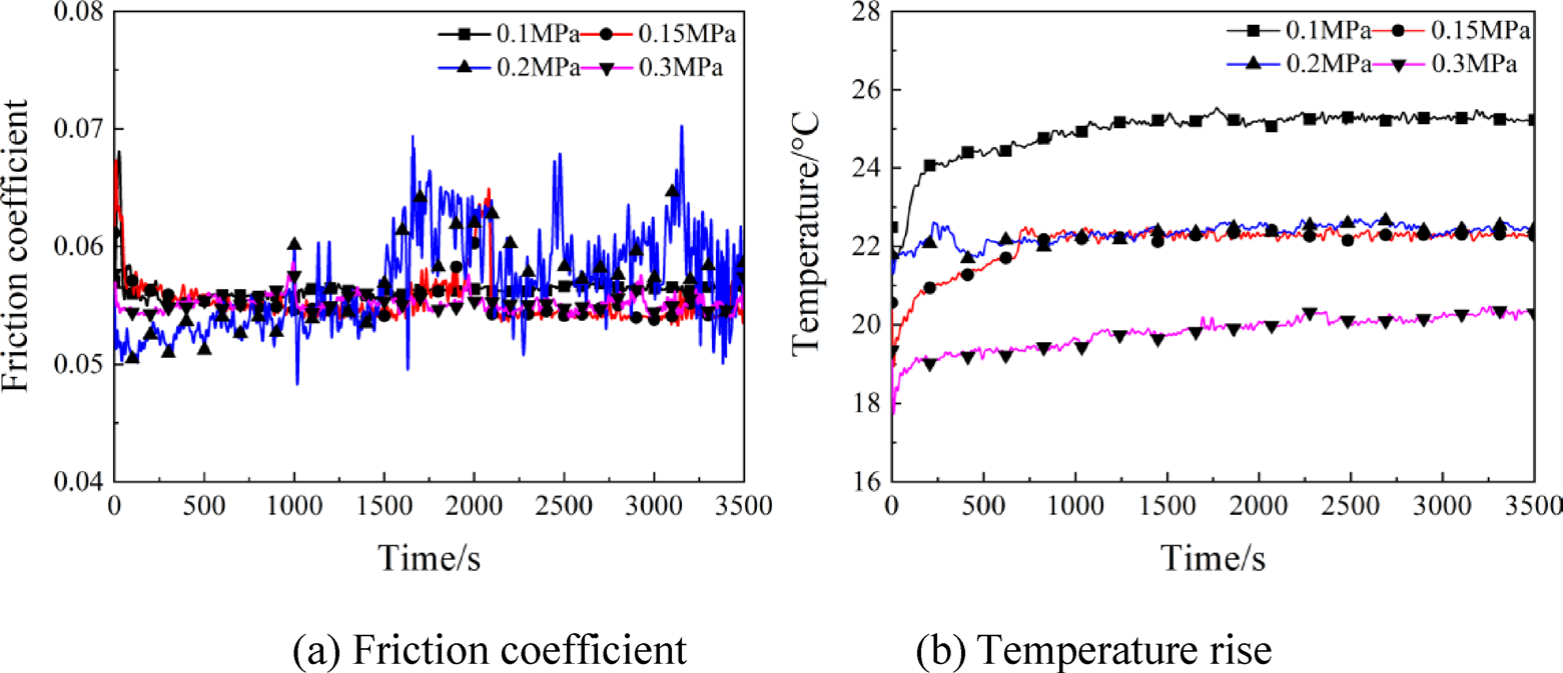
Effects of supply air pressure on the friction coefficient and temperature rise.

**Fig. 7:**
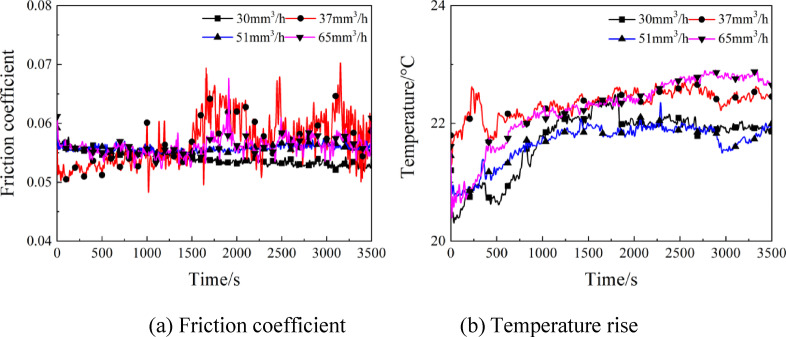
Effects of supply oil quantity on the friction coefficient and temperature rise.

The following Figs. 4, 5, 6 and  7 respectively show the effects of parameter changes on friction coefficient and temperature rise under oil air lubrication. The variation curves between friction coefficient and temperature rise are presented in Fig. 4 under various loads at rotating speed of 4000r/min. It is clear from Fig. 4(a) that acted by 10 N load, the friction coefficient of the contact parts undulated greatly within a range of 0 ~ 2000s, which then tended to stabilize, with a value of 0.052. As the load increased to 15 N, severer fluctuation of friction coefficient was noted, and the steady-stage friction coefficient was 0.055. When the load value reached 20 N, the friction coefficient presented downward trend instead, with a value fundamentally identical to that at 10 N. At load of 25 N, the friction coefficient showed little sway, and the steady-stage friction coefficient was the smallest, which was 0.05. The main reason is that under low load, the contact pressure is insufficient, and the oil film is difficult to form. When reaching a certain value, the oil film pressure increases, the contact surface separation is more thorough, and the friction coefficient decreases. Generally, the friction coefficient would increase with the enhancing load. After the load was large enough to exceed a specified value, the friction coefficient tended to stabilize or decrease with the further enhancement of load. As shown in Fig. 4(b), the temperature of friction pairs rose continuously with the passage of operating time at different loads. When the operating time was 2100s, the temperature generally peaked and then tended to be stable. At a load of 25 N, the maximum temperature was 25℃, showing a rise of 3℃ as compared to that at 10 N. This is attributed to the increased heat generation and reduced oil quantity of friction pairs caused by the increased load, which resulted in the worsening of service conditions.

Figure 5 displays the temporal variation curves of friction coefficient and temperature rise at various rotating speed under load of 15 N. As shown in Fig. 5(a), the friction coefficient decreased initially and then increased within a range of 0 ~ 700s, whereas decreased gradually to an eventual stability within a 700 ~ 2000s range. With the rotating speed increasing, the friction coefficient decreased from 0.065 to 0.055. With the increase of rotating speed, the dynamic pressure effect increases, the oil film gradually forms and completely isolates the contact surface, enters the fluid friction state, and the friction coefficient decreases. On the other hand, the increase of rotating speed heightened the entrainment speed and enlarged the oil film thickness, so that the friction coefficient decreased gradually until reaching stable state. According to Fig. 5(b), the temperature of friction pairs rose fastest in the initial stage, which stabilized in 1000s of operation fundamentally. As the speed changed between 2000 ~ 5000r/min, the temperature rose from 20℃ to 22.5℃. The reason is that with the heightening rotating speed, the rolling component of contact points between steel ball and disc increased, as well as the spin component of contact points, so that the tangential and sliding friction forces of the steel ball grew to cause increased heat generation, thereby intensifying the temperature rise.

In Fig. 6, the temporal variation curves of friction coefficient and temperature rise are presented under various supply air pressures at rotating speed of 4000r/min and load of 15 N. From Fig. 6(a), it is clear that at different supply air pressures, the friction coefficient was fundamentally stable at 0.055, suggesting little effect of the supply air pressure on the friction coefficient. According to Fig. 6(b), the friction pair temperature rose fastest in the initial stage, which stabilized within 1000s of operation fundamentally. In the initial stage, stable lubricating oil film was unable to be formed, thereby causing a dry friction phenomenon to lead to rise of the friction pair temperature. With the continuous replenishment of lubricating oil, stable elastohydrodynamic lubrication was formed, which improved the friction performance and stabilized the temperature in a gradual manner. As the supply air pressure changed from 0.1 MPa to 0.3 MPa, the temperature of friction pairs presented a downward trend. The maximum temperature of contact pairs was 25℃ at supply air pressure of 0.1 MPa, whereas was 20℃ at supply air pressure of 0.3 MPa. Regarding the reason, the compressed air took away most of the heat by convective effect when it flowed through the surface of contact pairs, which had a role in lowering the friction pair temperature. Enhancement of air cooling effect was noted with the increasing supply air pressure, and the temperature dropped accordingly.

The temporal variation curves of friction coefficient and temperature rise are displayed in Fig. 7 under various supply oil quantity at the rotating speed of 4000r/min and load of 15 N. As is clear from Fig. 7(a), the friction coefficient stabilized at 0.055 under different supply oil quantity, suggesting the insignificant effect of supply oil quantity on the friction coefficient. The reason is that the friction pairs were always in a full film lubrication state during the increase of oil supply from 30mm^3^/h to 65mm^3^/h, so that the reduction of friction coefficient was little influenced by the change of supply oil quantity. According to Fig. 7(b), the temperature of friction pairs rose gradually in the initial stage, which stabilized within 1000s fundamentally. The maximum temperature was 21.7℃ at supply oil quantity of 30mm^3^/h, whereas was 22.6℃ at supply oil quantity of 65 mm^3^/h. As the supply oil quantity changed from 30mm^3^/h to 65mm^3^/h, the disc sample surface was covered by excessive lubricating oil, which was detrimental to the heat dissipation and thereby resulted in a slight elevation of friction pair temperature.

### Analysis of wear scar morphologies

**Fig. 8:**
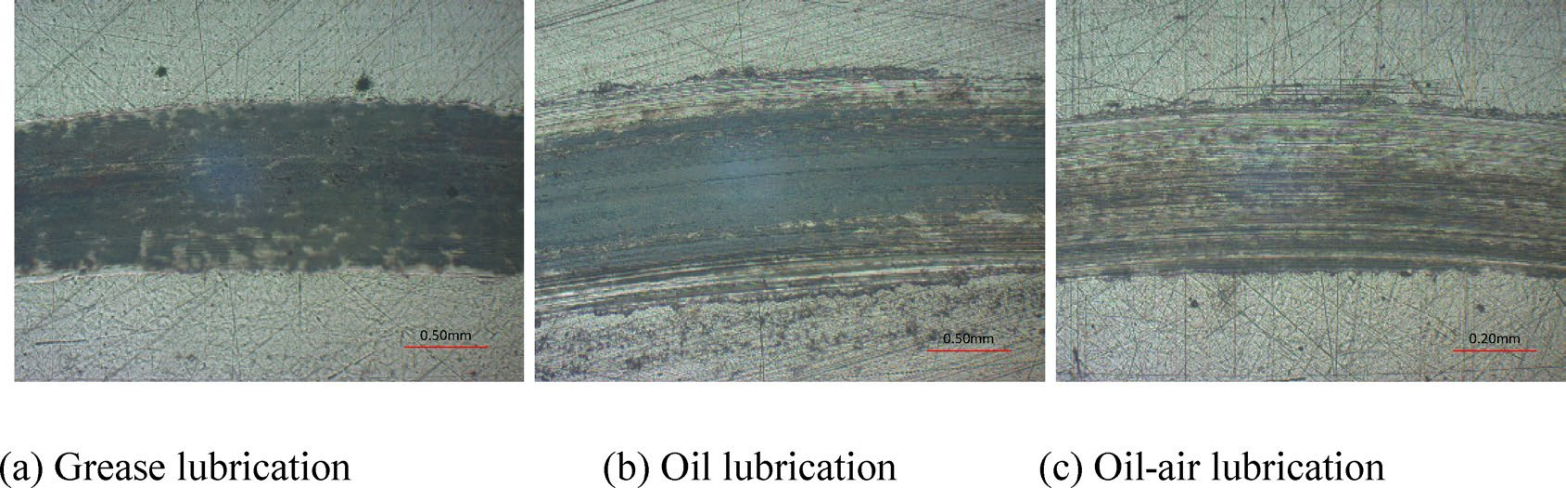
Wear scar surface morphologies with different lubrication modes.

**Fig. 9:**
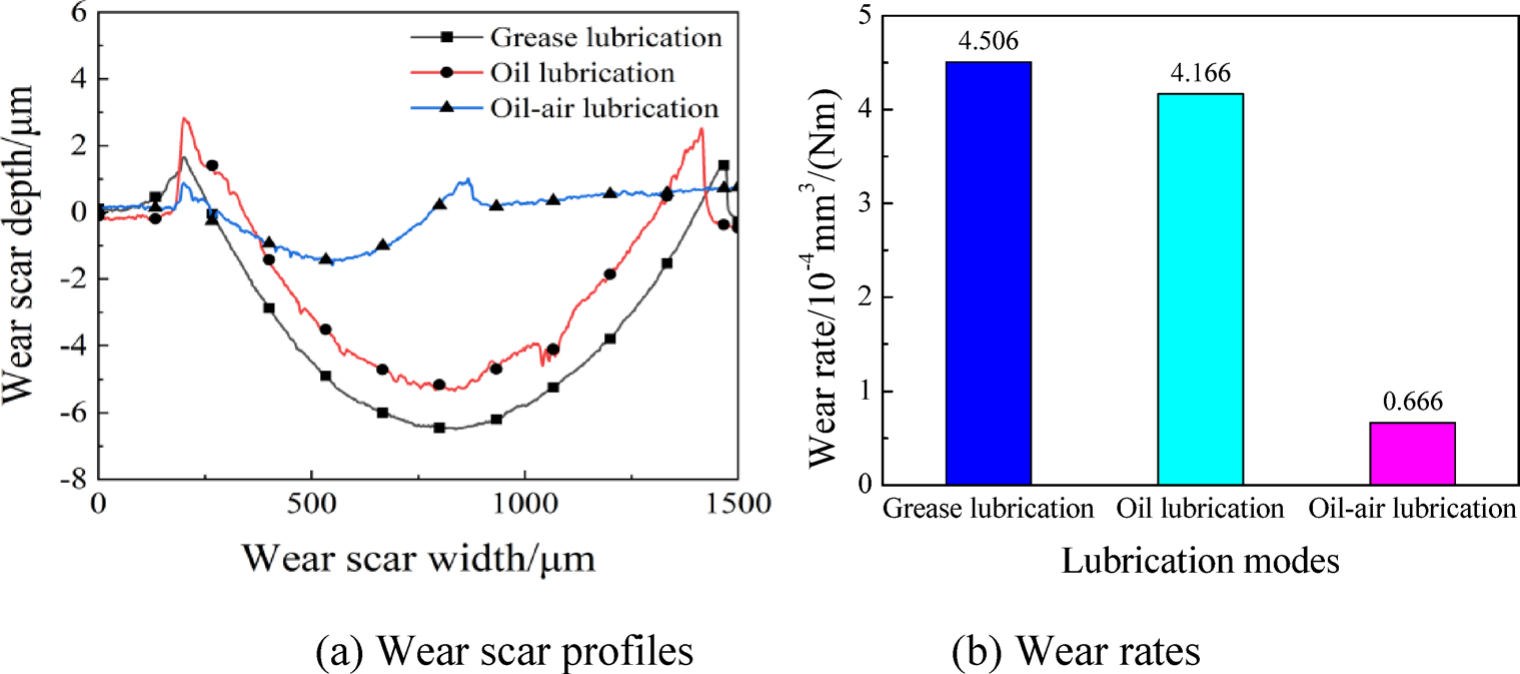
Wear scar profiles and wear rates with different lubrication modes.

Utilizing optical microscope, the micro-surface morphologies of wear scars were observed, and the wear rates were computed. Figures 8 and 9 illustrate the wear scar surface morphologies, profiles and wear rates with different lubrication modes. Clearly, the scar are black in color, mainly due to frictional heating, which causes metal oxidation discoloration. And it is most obvious under grease lubrication conditions, mainly due to the presence of organic molybdenum and other additives in Arcanol-L75 SPEED2.6 grease, which react with metals to form unstable products. The wear was rather severe under grease lubrication, with the width and depth of wear scar being 1270 μm and 8 μm, respectively. Under oil lubrication, the wear scar was 1210 μm and 8.3 μm in width and depth, respectively. In the case of oil-air lubrication, good lubrication performance was observed, the wear was slight, and the wear scar had width and depth of 660 μm and 2.6 μm, respectively. As shown in Fig. 9(b), the wear rates were 4.506 × 10^− 4^ mm^3^/(Nm) and 4.166 × 10^− 4^ mm^3^/(Nm) under grease lubrication and oil lubrication, respectively, while the wear rates under oil-air lubrication was 84% lower than the former two lubrication methods, with a value of 0.666 × 10^− 4^ mm^3^/(Nm) only. Regarding the reason, the lubricating grease was in a semi-solid state and had a poor fluidity, which was easily squeezed out of the contact zone to result in a dry friction state. Respecting oil lubrication, the lubricating oil content on the disc sample surface decreased gradually with the prolonging operating time, which deteriorated the lubrication of friction pairs. In contrast, the oil-air lubrication system offered continuous lubrication and cooling of contact surface. It achieved better lubrication efficiency when compared to the wear scar parameters in the grease and oil lubrication modes.

**Fig. 10:**
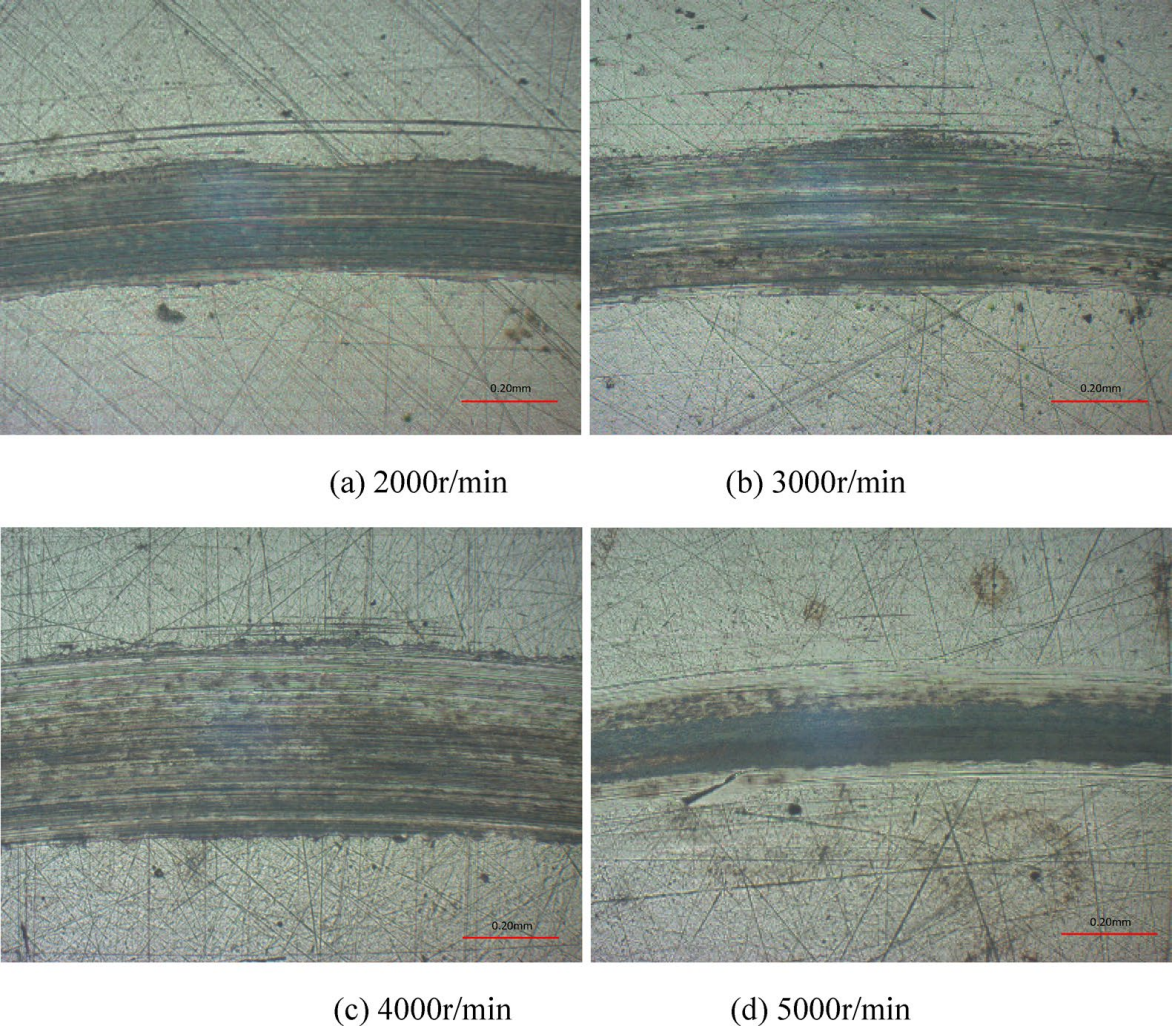
Wear scar surface morphologies at various rotating speeds with oil-air lubrication.

**Fig. 11:**
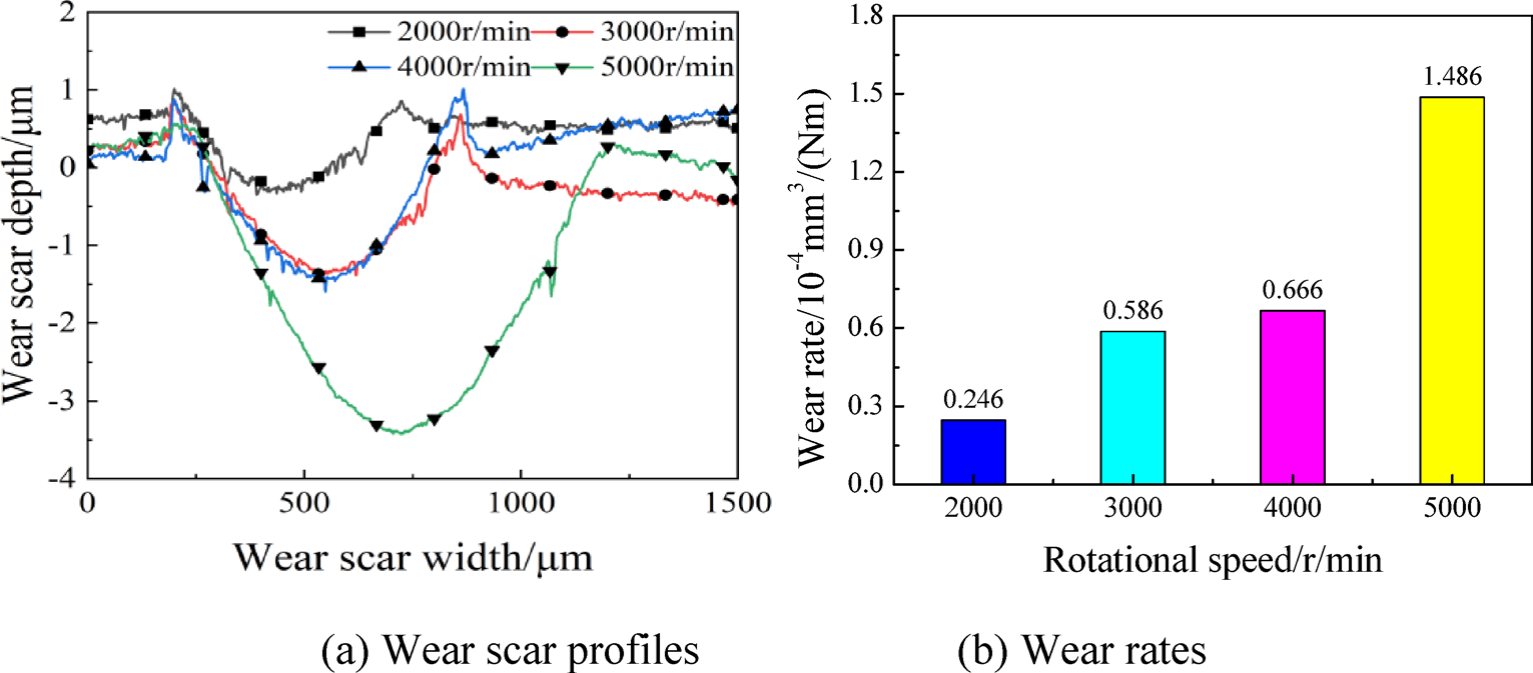
Wear scar profiles and wear rates at various rotating speeds with oil-air lubrication.

By analyzing the microscopic morphologies of wear scars under different lubrication modes, it is found that the wear scar profile changes were milder with the oil-air lubrication. In this lubrication regime, the rotating speed, load and supply oil quantity had certain influences on the friction and wear of contact pairs. The wear scar surface morphologies, profiles and wear rates are detailed in Figs. 10 and 11 under various rotating speeds. Clearly, at rotating speed of 2000r/min, the friction pairs had relatively good lubrication performance and slight wear, and the wear scar was 390 μm and 1.3 μm in width and depth, respectively. With the increase of rotating speed, the lubricating oil retained in the friction pairs diminished, and the friction frequency increased to cause rather severe wear. The wear scar had depth and width of 790 μm and 3.8 μm, respectively, at rotating speed of 5000r/min. As shown in Fig. 11(b), the wear rates presented an upward trend as rotating speed increased. With a value reaching 1.486 × 10^− 4^mm^3^/(Nm), the wear rates at 5000r/min was 83% higher than that at 2000r/min, which was 0.246mm^3^/(Nm), revealing a remarkable effect of rotating speed on the wear rates. This is attributed to the relatively short stay of lubricating oil on the friction pair surface due to the high rotation speeds. In view of the same duration of experiments, the number of frictions was larger at high rotating speed than that at low rotating speed, so that the wear was severer.

**Fig. 12:**
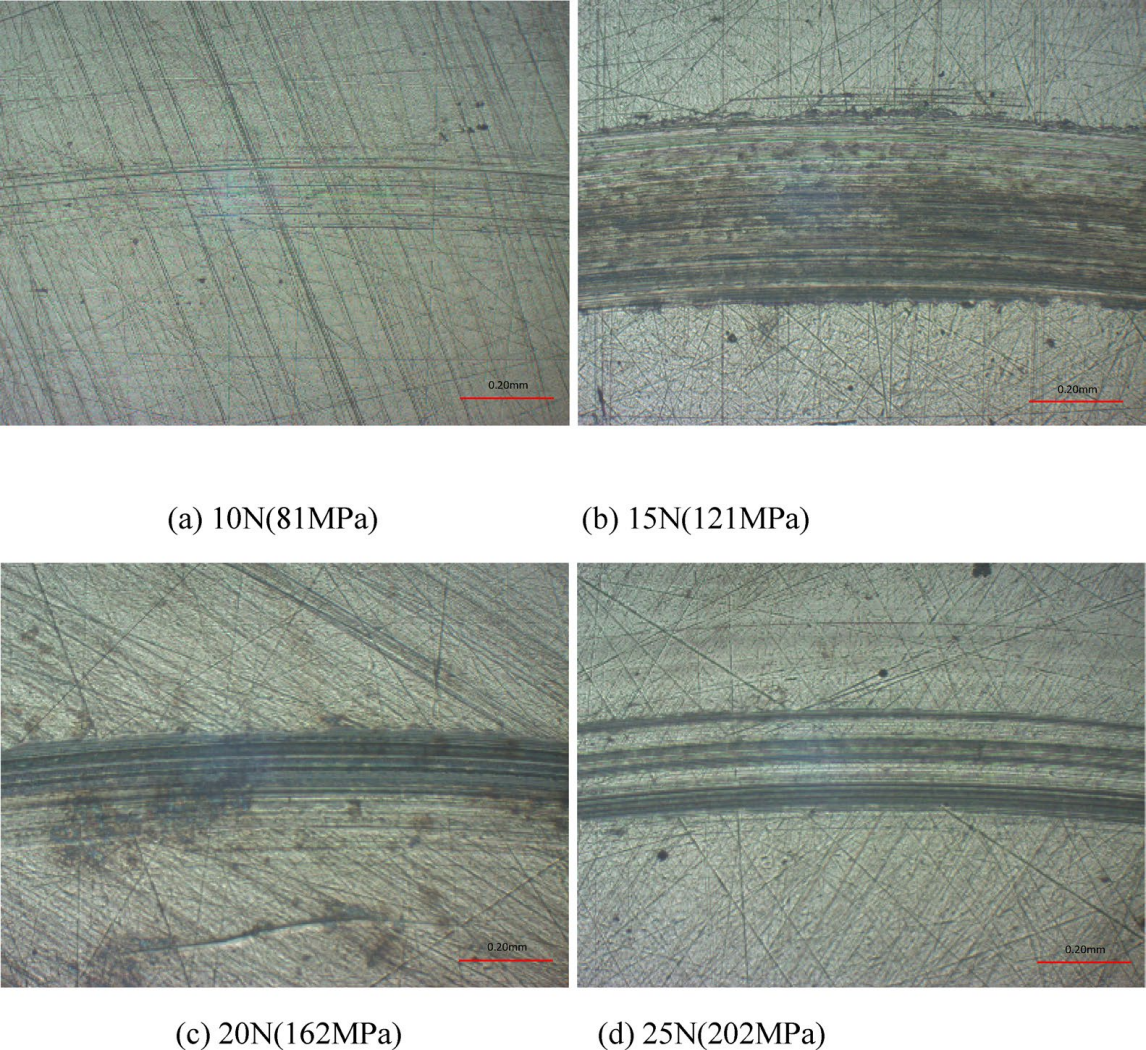
Wear scar surface morphologies at various loads with oil-air lubrication.

**Fig. 13:**
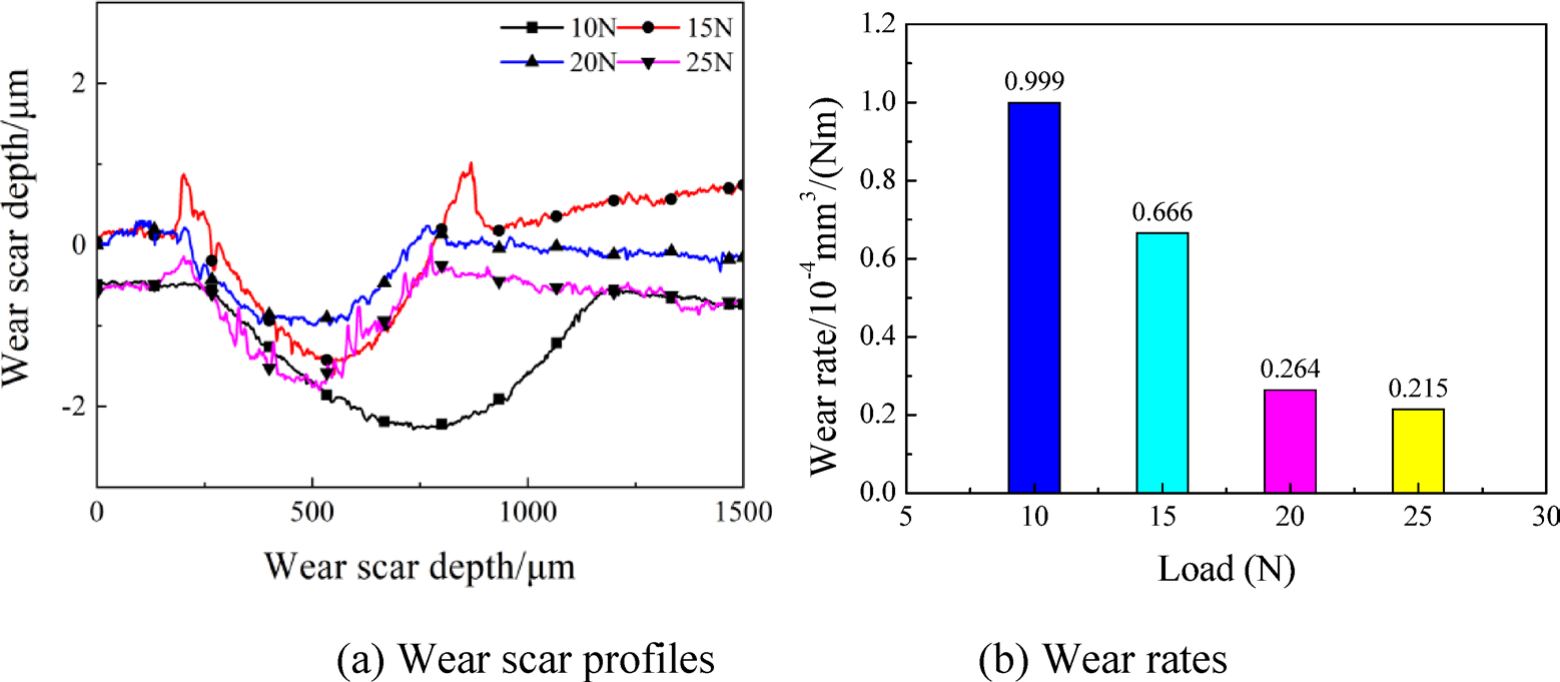
Wear scar profiles and wear rates at various loads with oil-air lubrication.

In Figs. 12 and 13, the wear scar surface morphologies, profiles and wear rates are presented at various loads. Clearly, the wear was slight at load of 10 N, and the wear scar had width and depth of 720 μm and 1.8 μm, respectively. This is attributed to the small frictional force of friction pairs caused by the low load. Table 5 lists the widths and depths of wear scars under different loads. With the enhancement of load, the wear scar depth increased initially and then decreased. According to Fig. 13(b), the wear rates declined with the increasing load at gradually decelerating rate. With a value of 0.215 × 10^− 4^ mm^3^/(Nm), the wear rates at 25 N was 78% lower than that at 10 N, which was 0.999 × 10^− 4^ mm^3^/(Nm). This is because the contact status of friction pairs was affected directly by the magnitude of load. Generally, the friction coefficient would increase with the enhancing load. After the load was large enough to exceed a specified value, the friction coefficient tended to stabilize or decrease with the further enhancement of load, while the wear status changed with the variation of friction coefficient.

**Table 5:** Width and depth of wear Scar applied by various load.

Load(*N*)	Wear scar width(µm)	Wear scar depth(µm)
10	720	1.8
15	530	2.6
20	420	1.8
25	470	1.4

**Fig. 14:**
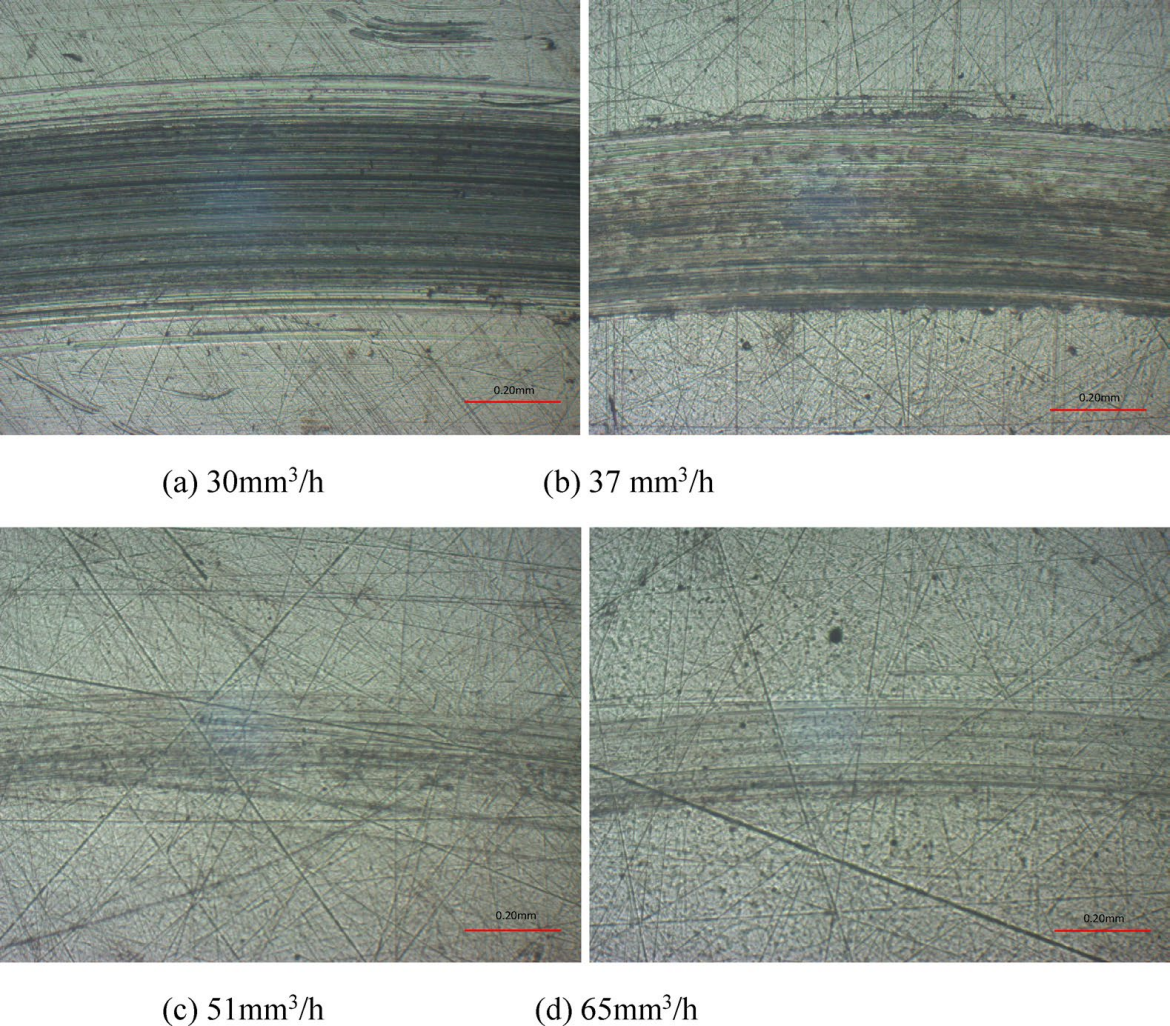
Wear scar surface morphologies at various supply oil quantities with oil-air lubrication.

**Fig. 15:**
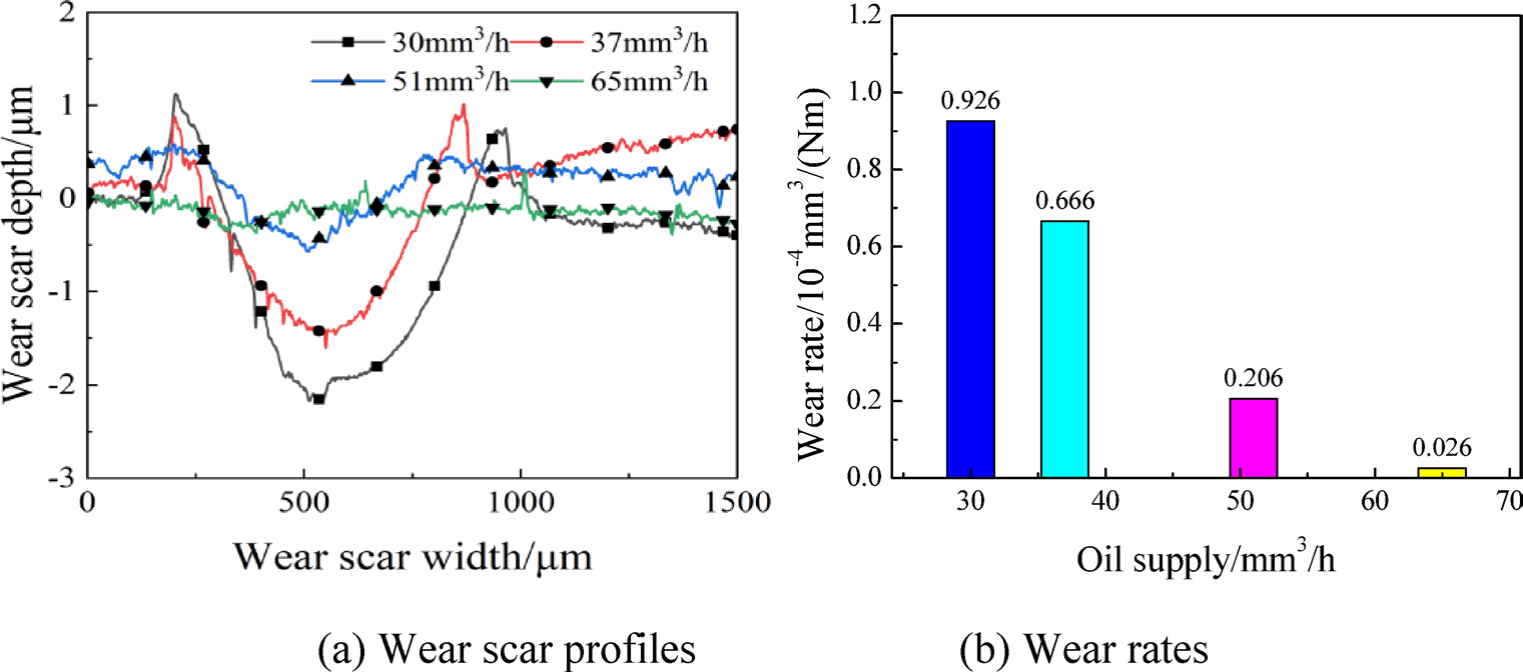
Wear scar profiles and wear rates at various supply oil quantities with oil-air lubrication.

Finally, the wear scar surface morphologies, profiles and wear rates are displayed in Figs. 14 and 15 at various supply oil quantity. As is clear, with the increase of supply oil quantity, the wear scars decreased in width and depth rapidly, where the scar width decreased from 750 μm to 290 μm, and the scar depth decreased from 3.41 μm to 0.35 μm. According to Fig. 15(b), the wear rates presented a downward trend with the increasing supply oil quantity. With a value of 0.026 × 10^− 4^mm^3^/(Nm), the wear rates at a 65mm^3^/h was 97% lower than that at a 30mm^3^/h, which was 0.926 × 10^− 4^ mm^3^/(Nm). Regarding the reason, the amount of lubricating oil at the contact pairs increased with growing supply oil quantity, so that the lubrication status was improved, and both the width and depth of wear scars were reduced.

## Conclusions

Rolling friction experiments of ball-on-disc contact pairs are conducted on a high-speed frictional wear test device, thereby investigating the effects of various operating condition parameters on the point contact friction coefficient, temperature rise and wear rates. Conclusions are drawn as follows:

1. Due to the continuous supply of compressed air and lubricant by the system, a good oil film is formed, effectively isolating the metal surface and reducing direct contact friction, resulting in a 29% to 0.055% reduction in friction coefficient and a 48% reduction in temperature rise to only 23 ℃ under oil-air lubrication.
2. Under oil-air lubrication, with the increase of rotating speed, the dynamic pressure effect increases, the oil film gradually forms and completely isolates the contact surface, enters the fluid friction state, and the friction coefficient decreases; when the load increases, the friction coefficient increases first and then decreases. The main reason is that under low load, the contact pressure is insufficient, and the oil film is difficult to form. When reaching a certain value, the oil film pressure increases, the contact surface separation is more thorough, and the friction coefficient decreases. As for the supply oil quantity and supply air pressure, they produce little effects on the friction coefficient.
3. With oil-air lubrication, the increase of load leads to the increase of contact stress, friction heat and temperature; the increase of rotational speed leads to the increase of friction frequency, heat generation and temperature. The increase of air supply pressure greatly inhibits the temperature rise of the friction pair and has a significant cooling effect.
4. At the same time, the speed of oil film formation lags behind the speed of motion, resulting in an increase in the proportion of boundary friction, an increase in the width and depth of wear traces, and an increase in the wear rate; the increase of oil supply can fully fill the friction pair gap, form a stable hydrodynamic oil film, reduce the width and depth of wear marks, and reduce the wear rate. Conclusively, oil-air lubrication is evidently superior to other lubrication modes for ball bearing.

## Data Availability

Data Availability StatementThe datasets generated and analyzed during the current study are not publicly available due to confidentiality agreements with industry partners. However, anonymized or processed data supporting the findings may be made available by the second author upon reasonable request, subject to approval by the relevant institution.
